# Economies of scale in constructing plant factories with artificial lighting and the economic viability of crop production

**DOI:** 10.3389/fpls.2022.992194

**Published:** 2022-09-08

**Authors:** Yunfei Zhuang, Na Lu, Shigeharu Shimamura, Atsushi Maruyama, Masao Kikuchi, Michiko Takagaki

**Affiliations:** ^1^Graduate School of Horticulture, Chiba University, Matsudo, Japan; ^2^Center for Environment, Health, and Field Sciences, Chiba University, Kashiwa, Japan; ^3^HANMO Co., Ltd., Kashiwa, Japan

**Keywords:** benefit-cost ratio, breakeven scale, lettuce, strawberries, transaction cost, urban agriculture

## Abstract

Since the introduction of LED lamps a decade ago, the plant factory with artificial lighting (PFAL) has been expected to be a savior that overcomes the food crisis, brings food safety, and enhances environmental friendliness. Despite such high expectations, the diffusion of commercial crop production in PFALs has been slow. It has been said that the main reason for this is the huge initial investment required to construct PFALs. This situation has attracted studies to access the economic feasibility of the crop production in PFALs. One thing strange in these studies is that they pay little attention to the scale of their PFALs. PFALs are factories so that they would be subject to economies of scale. If so, the scale of PFALs is an important factor that determines the economic feasibility of plant production in PFALs. However, no study has thus far attempted to examine whether economies of scale exist in the construction of PFALs. To fill this gap, this paper tries to examine, based on the data on the investment cost of PFAL construction collected from various countries and regions in the world, whether economies of scale exist in PFAL construction and, if yes, how it affects the economic viability of the plant production in PFALs by searching for the minimum scale that ensures PFAL crop production economically viable. The results show that economies of scale exist in PFAL construction, and that the production of lettuce, PFALs’ most popular crop, is now well on a commercial basis with the technology level of the most advanced PFAL operators, but strawberries has not reached that stage yet. It is also shown that crop production in PFALs is highly sensitive to changes in the yield and the price of the crops: A 30% decline either in the yield or the price of lettuce would easily bring PFALs bankruptcy. It is discussed that the optimum scale of PFALs would depend not only on the economies of scale but also on the transaction costs, such as the costs of searching and keeping a sufficient number of buyers who offer good and stable crop prices.

## Introduction

The plant factory with artificial lighting (PFAL), also called synonymously the vertical farm, controlled environment agriculture, and indoor agriculture, grows plants with artificial light in a shielded space like a factory. Although its origin may be traced back to the Hanging Gardens of Babylon in ancient times (Crumpacker, 2018), the PFAL today has only 20–30 years of history (Despommier, 2010; Kozai et al., 2022). Because of its high productivity, high resource use efficiency, high environmental gain, and characteristics that can be located in urban or semi-urban areas, crop production in PFALs has been expected to be an important solution to food security as well as food safety in the urban-overpopulated world in the 21st century (van Delden et al., 2021).

In the early stages of development, commercial production of crops in PFALs was going slowly, mainly because the initial investment needs were prohibitively high (Kozai, 2017). The emergence and introduction of LED lamps at the end of the last century drastically changed the situation (Bantis et al., 2018). It seems that the rapid progress in LEDs and the development of technology associated with the crop production in PFALs since the beginning of this century, especially since the early 2010s, have made the crop production, in particular leafy vegetable production, in PFALs a feasible commercial proposition (Benke and Tomkins, 2017; Pinstrup-Andersen, 2018; Shamshiri et al., 2018; Kozai et al., 2019, 2022).

On the other hand, however, harsh criticism against the PFAL still remains. As late as in 2020, an article appeared in an internationally renowned daily newspaper, questioning “Vertical farming: hope or hype?” (Terazono, 2020). It is pointed out in the article, referring to a Rabobank analyst in Netherlands as the source of information, that “Vertical farming occupies the equivalent of 30 hectares of land worldwide, …, compared with outdoor cultivation of about 50 m ha and 500,000 ha for greenhouses.” Asia, particularly in Japan, is the region where the commercial operation of PFALs has been going relatively better (Newbean Capital, 2016; Kozai et al., 2019; Harding, 2020). A large-scale survey of greenhouses and PFALs in Japan reveals that the number of PFALs under commercial operation had increased from 64 in 2011 to 197 in 2017 and has been stagnant since then (Japan Greenhouse Horticulture Association [JGHA], 2015-2022). It is estimated that the total cultivated area of these PFALs in 2021 is less than 60 ha, compared with 2 million ha of open upland field and 420,000 ha of greenhouse area in Japan. It could be said that, as in the world, the production of commercial crops by PFALs is hardly widespread even in Japan.

The gap between the hopeful future and the reality of commercial crop production in PFALs appears to be deep worldwide.

Certainly, such circumstances have induced studies on the economic feasibility of vegetable production in PFALs. For example, Eaves and Eaves (2018) and Avgoustaki and Xydis (2020) compare, respectively, the profitability of leafy vegetable production in a PFAL with that in a greenhouse with the conclusion that the PFAL production is more profitable, and the latter gives the internal rate of return to the investment in the construction of the PFAL as high as 35%. Lu et al. (2022) estimate the rate of return for constructing two different walk-in type mini PFALs: 61%/year and 22%/year, respectively. Another study by de Souza et al. (2022) also reports the internal rate of return of 14% for a PFAL. All these studies guarantee the optimistic prospect of PFAL production in terms of economic viability. One exception is Zeidler et al. (2017), which concludes that the production of lettuce and tomato in their PFAL has no economic advantage over the production in greenhouses.

It should be mentioned that the PFALs assessed by these studies are all fixed sizes, all different from small (the PFAL floor area of 3 m^2^: Lu et al., 2022) to large (2,625 m^2^: Zeidler et al., 2017). A plant factory is a “factory,” and in this respect, it is no different from a factory in any industrial sector. One of the most classical findings in industrial economics is that “factories (plants)” are subject to strong economies of scale (e.g., Moore, 1959; Haldi and Whitcomb, 1967). This means that the scale in the construction of PFALs could be an important factor in determining the economic performance of PFAL investments and operations. Almost all the literature on the PFAL, regardless of whether in favor of it or not, points out that the high initial investment cost is the most serious barrier to commercial crop production in PFALs. Though much less, many authors in the literature recognize that economies of scale exist in the construction cost of PFALs (e.g., Burton, 2019; Dahlberg and Lindén, 2019; Harding, 2020; Cambridge HOK, 2021; de Souza et al., 2022). None of them, however, gives any idea as to the degree of economies of scale in the construction of PFALs. If the construction cost of PFALs is subject to economies of scale, what scale of PFALs should we assume in assessing the economic viability of crop production in PFALs?

We have tried to find out some studies that attempt to estimate the economies of scale in PFAL construction but failed thus far. Shao et al. (2016) present a computational model to estimate the possible scale of PFALs for a given budget but give no information on the scale economies at all. In assessing the profitability of lettuce production in a PFAL, de Souza et al. (2022) provide two scenarios as to the scale of the PFAL, in addition to the base scenario, one if the scale is 50% and the other if it is twice as large. However, they assume constant return to scale in the PFAL construction: The capital cost of constructing the PFAL is assumed to be half in the former, and two times in the latter, of the base scenario. They succeed in detecting economies of scale in the current production of lettuce in the PFAL, but not in the PFAL construction.

To fill this gap, in this paper, we first try to estimate the degree of economies of scale in PFAL construction, using data on PFAL construction costs in Asia, North America, and Europe, and then we examine how the economies of scale affect the economic viability of crop production in PFALs, by estimating the minimum PFAL scale that brings about break-even in the crop production for a few typical crops grown in PFALs. This examination is expected to shed light on, and fill in, the deep gap that exists between the proponents in favor of, and critics against, the future prospect of commercial crop production in PFALs.

## Materials and methods

We first explain the methods we adopt in this study and then the data used in the analysis.

### Methods

#### Economies of scale

We are interested in whether investment costs to construct PFALs are subject to economies of scale. Haldi and Whitcomb (1967) find that plant investment costs in many industries are characterized by strong economies of scale. They measure economies of scale empirically as follows:


(1) K=a⁢Sb′′,

where K is the investment cost (US $) to construct a plant, S is the scale of the plant (in terms of output capacity; an example is the total ground area of the plant in m^2^), a′ is a constant, and the exponent b′ is a constant, called the “scale coefficient.” Since (dK/dS) (S/K) = b′, a value of b′ < 1 implies that, as the scale increases, the cost increases at a rate of increase less than that of the scale variable, hence increasing returns to scale, in other words, scale economies. Likewise, b′ = 1 means constant returns and b′ > 1 implies decreasing returns, or in other words, scale diseconomies.

In our study, we take the total plantable area (the total area of trays on which plants are grown; see sub-section 1-1 of Supplementary Material for the definition of this variable) of a PFAL as the variable to measure the scale of the PFAL and see sub-section 1-5 of Supplementary Material how different the degree of scale economies if we use PFALs’ building total floor are, instead of PFALs’ total plantable area.

Dividing through Eq. 1 by S, we obtain


(2) (K/S)=a⁢S(b′-1)′,o⁢r I=a⁢Ab′,

where A (= S) is the total plantable area (m^2^), I (= K/S = K/A) is the unit investment cost per plantable area (US $/m^2^), and b = b′ – 1. Note that b′ is a constant so is b. With this modification, the criterion for economies of scale becomes *b* < 0 for scale economies, *b* = 0 for constant returns, and *b* > 0 for scale diseconomies. We do use the unit investment cost for convenience to compare with the unit benefit obtained from the plant production in PFALs. Taking logarithm for both sides of Eq. 2, we obtain


(3) $$\mathrm{Ln}\,I=\mathrm{Ln}\,a^{\prime }+b\,\mathrm{Ln}\,A=\text{a}+b\,\mathrm{Ln}\,A,$$

where a = Ln a’. By estimating this equation by means of the regression method, the *t*-test as to the “scale elasticity” b will tell us whether the PFAL construction cost follows economies of scale.

The actual estimation of this equation is made with several additional variables that control the variances in the dependent variable “I” due to years, countries, and technology levels of sample PFALs. Of these variables, “years” (when sample PFALs were constructed, planned, or uploaded online) is a continuous variable, and all others are dummy variables.

#### The breakeven minimum scale of PFALs

First, let us define the benefit-cost (B/C) ratio of the investment in the construction of a PFAL in the annual flow term as follows:


(4) $$\text{B}/\text{C}={\text{P}}_{y}\text{Y}/\left\{\left[\sum_{\text{i}}{\text{P}}_{\text{i}}{\text{Input}}_{\text{i}}\right]+\left[\sum_{\text{j}}{\text{w}}_{\text{j}}{\text{Labor}}_{\text{j}}\right]+\left[\text{I}/\text{LS}\right]+\left[\text{αI}\right]+\left[\text{rI}\right]\right\},$$

where *B* = the benefit of the PFAL (US $/plantable area/year), *Y* = the quantity of the output produced and sold by the PFAL operator (kg/plantable area/year), *P*_*y*_ = the unit price (US $/kg) at which the output is sold (P_*y*_Y is called the revenue), *C* = the total cost of the PFAL (US $/plantable area/year), Input_*i*_ = the quantity of i-th current input used in the production per plantable area per year, *P*_*i*_ = the unit price of i-th current input, Labor_*j*_ = labor inputs (person-hours/plantable area/year) used in the production for j-th labor activity, *w*_*j*_ = the wage rate of type j labor (US $/person-hour), *I* = the unit investment cost of constructing the PFAL (US $/plantable area), LS = the lifespan of the PFAL (years), α = maintenance costs of the PFAL (in % share of the total investment), and *r* = the interest rate (%/year). Note that the benefit and cost are all defined in terms of “per plantable area.”

At B/C = 1, *B* = C, so that no economic loss. Set B/C = 1 and solve the equation for I,


(5) I*=(Py⁢Y-∑iPi⁢Inputi-∑jwj⁢Laborj)/(1/LS+α+r)=Surplus/(1/LS+α+r),

where I* is the (breakeven) investment that ensures no economic loss in producing the crop in question, and Surplus = (P_y_Y−∑_i_P_i_Input_i_−∑_j_w_j_Labor_j_). Note that “Surplus” is the profit in the current crop production, with no regard of tax and subsidy. Also note that Eq. 5 requires the following conditions:


(6) $$I^{*}\geq 0\,a\,n\,d\,S\,u\,r\,p\,l\,u\,s\geq 0.$$

Solving Eq. 3 in the previous sub-section with respect to A, we obtain the following equation:


(7) A=Exp⁢[(Ln⁢I-a)/b],

Inserting I* into Eq. 7, we obtain


(8) A*=Exp⁢{⟨Ln⁢[Surplus/(1/LS+α+r)]-a⟩/b},

A*, thus estimated, is the scale of PFAL that ensures neither economic loss nor profit in producing the crop in question.

#### Crops to be examined

As crops for which the economically viable minimum scale is examined, we select two crops: lettuce and strawberry.

Leafy vegetables are the most popular crops commercially grown in PFALs worldwide, of which lettuce dominates taking an overwhelmingly large share. The large-scale PFAL surveys in Japan, mentioned earlier, show that more than 90% of commercially operating PFALs have been growing leafy vegetables, and more than 90% of which are made up of lettuce (Japan Greenhouse Horticulture Association [JGHA], 2018, 2019, 2020, 2021, 2022). Lettuce is the first crop to be examined in the context of this paper.

Strawberry is a fruit vegetable. Tomato is the most preferred fruit vegetable in greenhouses and solar-using plant factories, followed by strawberry (Japan Greenhouse Horticulture Association [JGHA], 2015-2022; Costa and Heuvelink, 2018). According to the PFAL surveys, no PFAL operator in Japan had dare tried to grow any fruit vegetable until 2020. In 2021, however, a PFAL operator began to grow strawberry. It is eagerly expected that strawberry is soon added to the list of crops stably produced in PFALs (Kozai et al., 2018; O’Sullivan et al., 2020, Nikkei, 2021, SPREAD, 2021). Strawberry is thus worth to be examined its breakeven minimum scale of PFALs.

### Data requirements and data collection

We need three groups of data in this paper. First, data on the unit investment cost, I, and the total plantable area, A, of as many PFALs as possible, to estimate Eq. 3. Second, data on the revenues, costs, and “surplus” in current production of lettuce and strawberry in PFALs, required to estimate I*, the breakeven investment, in Eq. 5 and A*, the breakeven scale of PFALs, in Eq. 8. Third, data on the lifespan of PFAL facilities, the maintenance cost rate, and the capital interest rate in Eqs 5 and 8.

For the first group, we collect data through searching the internet for sites that contain information on PFAL construction costs; monographs, journal papers, survey/project reports, homepages of various organizations, advertisements, online shops, etc. In addition, we design six PFALs of different scales and estimate their construction costs, based on our experiences in designing PFALs.

For the second and the third groups, we obtain necessary data by reviewing the past studies and surveys, including our own data.

## Results

### Estimation of economies of scale in PFAL construction

#### PFAL construction cost

We have collected data for 26 PFALs from 14 sources, including ours, for which the necessary data, i.e., the unit investment cost per plantable area (I) and the total plantable area (A), are duly available (Table 1). The data sources of these PFALs are presented in Supplementary Table S1.

**TABLE 1:** List of sample PFALs included in this study, in the order of the scale of PFALs’ total plantable area.

PFAL ID	Total plantable area^a^	Cultivation-zone floor area^b^	PFAL building floor area	Planting rack tiers	Total construction cost	Unit construction cost	Technology level^c^	Year^d^	Country	Source ID^e^
										
	(1) = (2)*(3)	(2)		(3)	(4)	(5) = (4)/(1)				
	m^2^	m^2^	m^2^		US $ 000	US $/m^2^				
1	100,000	10,000	19,491	10	115,891	1,159	High	2020	United States	1
2	26,013	2,008	6,410	13	39,000	1,499	High	2019	United States	2
3	20,000	1,250	2,625	16	32,635	1,632	High	2015	EU	3
4	4,000	1,000	2,500	4	2,673	668		2011	Japan	4
5	3,780	315	930	12	6,295	1,665		2021	Japan	5
6	3,600	360	1,800	10	2,727	758		2019	Japan	6
7	3,528	321	1,000	11	7,136	2,023	High	2017	Japan	7
8	3,500	292	1,319	12	4,545	1,299		2013	Japan	6
9	3,380	338	676	10	3,591	1,062		2010	Japan	8
10	2,520	315	930	8	5,418	2,150		2021	Japan	5
11	2,184	364	1,000	6	3,208	1,469		2016	Japan	9
12	1,500	167	782	9	3,182	2,121	High	2018	Japan	6
13	1,370	274	391	5	2,500	1,825		2021	Canada	10
14	1,300	130	498	10	2,364	1,818		2013	Japan	11
15	1,225	175	350	7	1,400	1,143		2013	Japan	12
16	1,050	88	357	12	2,007	1,911		2021	Japan	5
17	700	88	357	8	1,744	2,492		2021	Japan	5
18	525	44	165	12	1,209	2,304		2021	Japan	5
19	400	100	417	4	1,364	3,409	High	2016	Japan	6
20	350	44	165	8	1,051	3,004		2021	Japan	5
21	134	22	28	6	145	1,080	Low	2021	Canada	10
22	112	22	28	5	100	893	Low	2021	China	13
23	89	22	28	4	237	2,652		2021	Canada	10
24	61	20	28	3	160	2,609		2021	Canada	10
25	52	10	15	5	60	1,152	Low	2020	China	14
26	12	3	3	4	50	4,167		2020	Japan	14

^a^The summation of the area of planting trays. For definitions of this and other variables, see sub-section 1-1 of Supplementary Material.

^b^The total floor area taken by planting racks = the total bottom area of planting racks.

^c^The level of technology related to the systems of hydroponic production, environment control, and automation of cultivation works, adopted by PFALs. Three levels are distinguished: high (highly advanced, highly automated), average, and low (more primitive, more dependent on manual labor). The PFALs with high technology are those so announced in the data sources. PFALs with low technology are partly those so announced in the sources and partly so identified by the authors. The PFALs, for which this column is blank are of the average technology or those with no information on the technology level.

^d^The year the PFAL was constructed or designed, or its information was uploaded online.

^e^IDs showing data sources, which are explained in Supplementary Table S1.

It should be noted that this main dataset is not without problems. Firstly, the dataset consists of data of very different quality. Some data sources give very accurate and highly detailed estimates of the construction costs, while others do give very rough data only. Secondly, the confidentiality of technology and patent information, tax consideration, etc., not only limit the cases where PFAL construction costs are disclosed, but even if information is available, it might possibly give certain undesirable biases to the information disclosed. Thirdly, the dataset includes PFALs constructed and those at design level. The design-level PFALs, though deliberately designed for good performances not only engineeringly, biologically and environmentally but also economically, some revisions might be required if actually built. The number of observations of this dataset, 26, may or may not be sufficient to cancel out biases and errors, if any, caused by these problems. In any case, we should be aware of these problems when interpreting the results.

The 26 sample PFALs in Table 1 are from five different countries and regions, with relevant years ranging from 2011 to 2021. The construction costs in the table are shown in terms of the current United States dollars, converted from respective local currencies using the exchange rate in respective years. Since the value of the United States dollar has fluctuated little during this period and the use of United States dollars in constant prices has altered the results only slightly without changing any of our conclusions, we use the cost data in United States dollars in current prices throughout this paper.

#### Economies of scale

**FIGURE 1:**
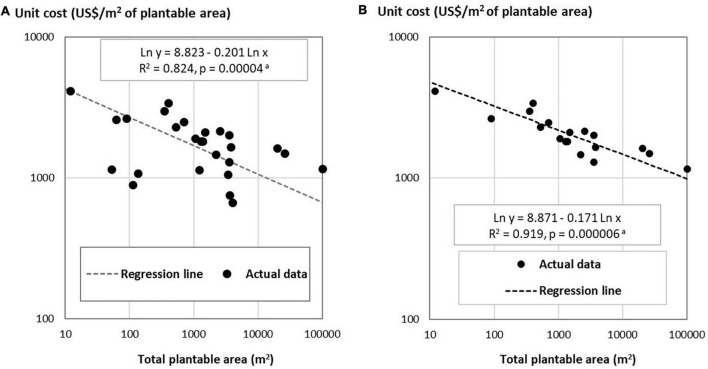
The relationship between the unit cost of PFAL construction and the scale of PFAL’s total plantable area: panel **(A)** for all sample PFALs (*n* = 26) and panel **(B)** for above-average PFALs (*n* = 18). ^a^For each chart, the regression line drawn is obtained by inserting the respective means to the seven variables from “Year” to “Canada” of Regression #1 **(A)** and Regression #2 **(B)** in Table 2. The probability shown is the probability that the null hypothesis of no slope is accepted.

Data on the unit investment cost, I, and the total plantable area, A, in Table 1 are depicted in Figure 1A and the results of regression estimation of Eq. 3 are presented in Table 2. The dataset reveals significant economies of scale, with the scale elasticity of – 0.201 (Regression #1 in Table 2). The dotted line drawn in Figure 1A is the Regression #1, the intercept term of which is adjusted for by evaluating seven non-slope variables from “Year” to “Canada” at their respective means.

**TABLE 2:** The results of regression analyses applied to the PFAL construction cost data: Regressing Ln (Unit construction cost) on Ln (Plantable area), year and other dummy variables.^a^

Regression number	Regression #1		Regression #2		Regression #3	
	For all PFAL samples (*n* = 26)^b^		For PFALs above average (*n* = 18)^c^		For PFALs below average (*n* = 8)^d^	
	Coeffi.	Prob.	Coeffi.	Prob.	Coeffi.	Prob.
Ln (Plantable area)	**–0.201**	**8.3E–05**	**–0.171**	**5.9E-06**	**–0.283**	**0.001**
Year	**0.048**	**0.008**	**0.030**	**0.028**		
High-Tech PFAL	**0.426**	**0.011**	**0.278**	**0.005**		
Low-Tech PFAL	**–0.844**	**0.006**			**–0.806**	**0.005**
China	-0.345	0.319				
EU	0.243	0.413	0.125	0.417		
United States	0.003	0.992	–0.061	0.669		
Canada	–0.192	0.267	–0.169	0.105		
Intercept	**–87.7**	**0.014**	–50.8	0.054	**9.03**	**1.2E–06**
*R* ^2^	0.824		0.919		0.890	
Degree of freedom	17		11		5	

^a^The estimation results of Eq. 3, with the following additional explanatory variables to control “errors and biases.” The regression equation to be estimated for the full model of Regression #1 is as follows:

Ln (Unit construction cost) = a + b Ln (Plantable area) + c_1_ Year + c_2_ High-Tech PFAL + c_3_ Low-Tech PFAL + c_4_ China + c_5_ EU + c_6_ United States + c_7_ Canada, where Year = the year the PFAL was constructed, designed, or referred to; High-Tech PFAL = a dummy variable taking 1 if a sample PFAL is said to be with high-technology and 0 if not; Low-Tech PFAL = a dummy variable taking 1 if a sample PFAL is said to be with low-technology and 0 if not; China, EU, United States, and Canada are all dummy variables taking 1 if a sample PFAL is from China, EU, United States, or Canada, respectively, and 0 if not; and a, b, and c_1_–c_7_ are regression coefficients to be estimated. The base country for the country dummy variables is Japan.

In the table, “Coeffi” stands for regression coefficients estimated and “Prob.” for the provability that the null hypothesis that the estimated regression coefficient is not statistically different from 0 is accepted. The probability that is smaller than 0.001 is shown in the index form, e.g., 8.3 E-05 = 8.3 × 10^–5^. The coefficients that are shown in bold letters are statistically significant at *p* < 0.05.

^b^*R*^2^ of the simple regression (*n* = 26) is 0.160 (*p* = 0.0428).

^c^For the 18 PFALs which are located on and above the regression line in Figure 1A. The PFALs “on” the regression line are defined as those which are located below the regression line but within the neighborhood less than US$ 100/m^2^ in the vertical distance. *R*^2^ of the simple regression (*n* = 18) is 0.754 (*p* = 3.0E-06).

^d^For the 8 PFALs which are located below the regression line in Figure 1A. Because of the small degree of freedom, the regression equation shown is the only one which gives significant results. *R*^2^ of the simple regression (*n* = 8) is 0.374 (*p* = 0.107).

Among the 26 sample PFALs, the cost differentials for PFALs of similar scale are large (Figure 1A). At the scale around 100 m^2^, the differential in the unit cost is as large as US $ 1,700/m^2^ between high cost PFALs and low cost PFALs. Similarly, at the scale around 3,500–4,000 m^2^, the unit cost differentials are about US $ 1,300/m^2^.

The significant positive regression coefficient of “Year” in Regression #1 in Table 2 suggests that there is a tendency that the unit construction cost of PFALs in recent years is higher than in earlier years. The significant positive coefficient of “High-tech PFAL” dummy and the significant negative coefficient of “Low-tech PFAL” dummy also suggest that the unit construction cost reflects difference in the technology levels embodied in PFALs. The type of PFAL, such as shipping-container PFAL or ordinary-factory-type PFAL, may also affect the unit-construction cost.

For example, there are three PFALs at the scale around 100 m^2^ far below the regression line (Figure 1A). These PFALs are of shipping container type, one is using a 20-foot container and the other two each using a 40-foot container. One of these 40-foot container type PFALs, you can buy in an online shop. These are indeed low-cost PFALs, as you may conceive (Alberta Agriculture and Forestry, 2017; Alibaba.com, 2022; Lu et al., 2022). It should be noted, however, that shipping-container type PFALs are not always cheap. There are two PFALs of similar scales, straight above these three PFALs in Figure 1A, just on or only slightly below the regression line. These two are of the 40-foot container type, with some advanced technology (Alberta Agriculture and Forestry, 2017).

At the scale around 3,500–4,000 m^2^, there are two PFALs, located far below the regression line. One of them is a PFAL, which was planned as early as 2011 by a city government in Japan but ended up unsuccessfully. Straight above this PFAL at a similar scale, three PFALs are found lining up along the top frontier line or just below it. One of them is a PFAL operated by one of the largest PFAL company in Japan well known for its successful vegetable production (Hayashi, 2020). The other two are among those we designed, which could attain the best crop growing performance within the purview of the present PFAL technology with minimum automation.

These observations indicate that the PFALs located below the regression line tend to be those with relatively simpler technology, whereas those on and above the line are of relatively better technology. The regression estimations applied to 18 above-average PFALs and 8 below-average PFALs both show reasonably good fitting, but *R*^2^ of Regression #2 for the above-average PFALs is as high as 0.919, with the sample PFALs that are less scattered, centering around the regression line, as shown in Figure 1B. The partial *R*^2^ of the scale variable (“Ln Plantable area”) is estimated 0.916 (not shown in Table 2), i.e., more than 99% of the *R*^2^ (0.916/0.919) of this regression equation is accounted for by the variance in the scale variable in log transformation. It is rather surprising that the variation in the scale of PFAL alone accounts for nearly 100% of the variation in the unit construction costs of PFAL in different countries explained by the explanatory variables. We will use Regression #2 for the above-average PFALs as the main equation to estimate the economically viable minimum scale of PFALs, with Regression #1 as a reference equation (for a supplementary analysis to check if the PFALs in the main dataset aptly belong to the PFAL population, see sub-section 1.4 of Supplementary Material).

### The economically viable minimum scale of PFALs

We estimate the minimum scale of PFALs for lettuce and strawberry, using Eq. 8.

#### Crop performance in PFALs and other assumptions

For the second and the third data groups, we obtain necessary data by reviewing the past studies and surveys, including our own data. The crop performance of lettuce and strawberries in PFALs are assumed as shown in Table 3.

**TABLE 3:** Assumed levels of lettuce and strawberry production in PFALs and their production costs.^a^

		Lettuce	Strawberries
Plant/fruit weight (1)	g/plant	180	800
Planting density (2)	no. of plant/m^2^	80	10
Crops harvested per year (3)		8	2.5
Yield per year (4) = (1)*(2)*(3)/1000	kg/m^2^/year	115	20
Harvest loss (5)	%	5	5
Output selling price (6)	US$/kg	11	50
Revenue (7) = (4)*(1–(5))*(6)	US$/m^2^/year	1,204	950
Current production costs			
Labor	US$/m^2^/year	279	175
Electricity	US$/m^2^/year	166	199
Seeds and nutrients	US$/m^2^/year	86	201
Water and others	US$/m^2^/year	12	12
Packaging and logistics	US$/m^2^/year	157	205
Total (8)	US$/m^2^/year	700	791
Surplus (9) = (7)–(8)	US$/m^2^/year	**504**	**127 ^b^**

^a^For details of the estimation and sources of the data, see the second section of Supplementary Material.

^b^Adjusted for the 20% reduction in the planted area due to the labor work setting for strawberry production.

The yield of lettuce is assumed to be 115 kg/m^2^/year. This is a high yield, which used to be challenging to attain several years ago. Thanks to the rapid improvements in LED in recent years, developments of new lettuce varieties suited to the production in PFALs, and PFAL operators’ efforts to improve crop growing technology, the yield of lettuce has increased dramatically in the last 5 years or so. The assumed yield level is the level that PFAL operators with advanced growing technology can attain stably (for details, see sub-section 2.1 of Supplementary Material).

The yield of strawberry is assumed to be 20 kg/m^2^/year. This is the highest yield level attained in PFALs by researchers in their experiments. The kind of yield revolution, which has happened to lettuce, seems not to have happened yet to strawberry. Unlike in the case of lettuce, we have failed to find out data on the revenue-cost structure of strawberries cultivation in PFALs. We estimate it by referring to the cases of lettuce and other fruit vegetables. Another handicap for strawberry to be grown in PFALs is that its cultivation requires more space than lettuce cultivation for the needs of labor works (for details, see sub-section 2.2 of Supplementary Material).

It should be noted that no subsidy as well as no tax are assumed in the production costs. Taxes are not included because the rates of taxes differ across countries. Subsidies are not included because we are interested in the economic viability of crop production in PFALs without any subsidy. A more important note to make is that no scale economies are assumed in the production costs for lettuce and strawberry production. It is assumed that all cost items are divisible and freely variable without any fixed factor. The production costs could be subject to economies of scale because of such fixed factors as permanent employees paid with monthly or yearly salaries. We make this assumption for the sake of simplicity in order to focus on the scale economies in PFAL construction.

Equation 8 requires the lifespan of PFAL building and other durable facilities (LS), the rate of maintenance expenses (α), and interest rate (r). We assume LS = 15 years, α = 1.5%, and *r* = 5% (see section 3 of Supplementary Material for more details in these assumptions).

#### PFALs’ breakeven minimum scales

The estimated breakeven scales are presented in Table 4 for the two crops for the above-average PFALs (Figure 1B) and for the average PFALs (Figure 1A).

**TABLE 4:** The minimum PFAL scale (total plantable area) at which the break-even is brought about in the commercial vegetable production: lettuce and strawberries.^a^

	Lettuce		Strawberries	
The above-average PFALs^b^	38	m^2^	115,697	m^2^
The average PFALs^c^	17	m^2^	16,131	m^2^

^a^Estimated as A* in Eq. 8 in the text. The surplus in lettuce and strawberries production are given in Table 3. The assumed levels of LS (life span of PFALs), a (the ratio of maintenance expenditures to the total investment), and r (interest rate) are 15 years, 1.5%, and 5%, respectively. For details, see the third section of Supplementary Material.

^b^Based on Regression #2 in Table 2, as depicted in Figure 1B.

^c^Based on Regression #1 in Table 2, as depicted in Figure 1A.

##### Lettuce

The above-average scale equation gives the scale of 38 m^2^ as the breakeven scale. Investing in setting up a PFAL of this scale for lettuce cultivation, you would attain breakeven in your business of lettuce production. The corresponding breakeven investment cost, I* (Eq. 5), is computed as US $ 3,821/m^2^, so that the total investment amounts to US $ 145,198 per PFAL. Suppose your planting rack is 5 tiers, then the cultivation-zone floor area in this case is 7.6 m^2^. This cultivation-zone floor area, in turn, leads to the PFAL building floor area of about 11 m^2^, if we assume that the cultivation-zone area takes up 70% of the building floor area (see sub-section 1.4 of Supplementary Material). This scale is close to the scale of PFAL #25 in Table 1, which is a 20-foot shipping container type PFAL.

Any PFAL of the scale larger than this breakeven scale gives a positive profit to the operator who grows lettuce. Suppose you invest in constructing a PFAL of 3,000 m^2^ of total plantable area (the average scale of commercially operating PFALs in Japan in 2021: Japan Greenhouse Horticulture Association [JGHA], 2015-2022), the unit investment cost of the above-average PFAL of this scale is US $ 1,808/m^2^ and the B/C ratio (Eq. 4) is calculated as 1.28, i.e., the annual rate of return of 28%. If we apply the average scale equation (Figure 1A), the breakeven scale is reduced to 17 m^2^. This scale is a bit larger than 12 m^2^ of PFAL #26 (Table 1). On this average scale equation, the rate of return for the investment to construct a PFAL of 3,000 m^2^ increases to 37% per annum.

Whichever is the case, it is evident that lettuce has established a solid status as a commercially cultivated crop for PFAL operators who attain the level of the revenue-cost performance in lettuce production specified in Table 3.

##### Strawberries

The minimum scale for strawberry production in PFALs is estimated to be 115,697 m^2^ if the above-average scale equation is applied and 16,131 m^2^ if the average scale equation is applied. The former scale is larger than the scale of the largest PFAL that has ever been conceived (PFAL #1 in Table 1; Asseng et al., 2020). Even the latter scale is very large if compared to the average scale of commercially operating PFALs. There would be few people who dare want to construct an “above-average” PFAL of a scale greater than the breakeven minimum scale to grow strawberries under the given conditions. You may want to construct an “average” PFAL of the scale larger than the breakeven scale to obtain a positive profit from strawberry production, but the guaranteed annual rate of return on the investment to build a 50,000 m^2^ PFAL is estimated to be mere 3.5%. These results suggest that strawberry is still premature to be a crop grown in PFALs on a commercial base.

### Sensitivity tests

How the breakeven minimum scale changes as one of the assumed parameters changes? We present the results of sensitivity tests conducted for “the above-average PFALs” to examine how the minimum scale changes, for lettuce, if the revenue or the labor cost changes, and for strawberries, if the revenue or the electricity cost changes. Since revenue = unit yield × unit output price, a change in revenue by, say, 10%, means either of unit yield or unit price changes by 10% while the other remains constant. Likewise, a positive change in labor cost or in electricity cost means that either the input requirement of labor or electricity increases while the corresponding price remains constant or the price (the wage rate or the electricity charge) changes while the corresponding input requirement remains constant.

**FIGURE 2:**
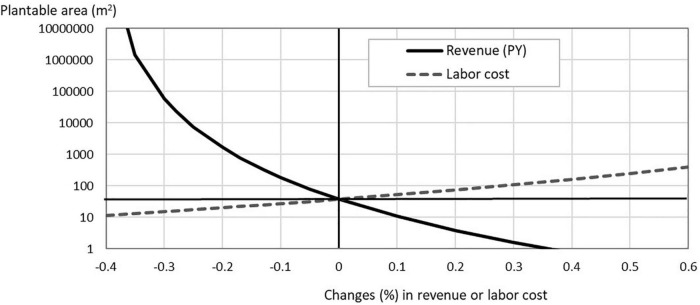
Sensitivity analyses for lettuce production in PFALs: How the minimum plantable area, which satisfies (B/C) = 1 in lettuce production, changes when the revenue (PY) or labor cost changes (for the above-average PFALs). The starting point before the change is *the minimum plantable area = 38 m^2^, Y = 115 kg/m^2^/year, P = US$ 11/kg, and labor cost = US$ 279/m^2^/year*.

**FIGURE 3:**
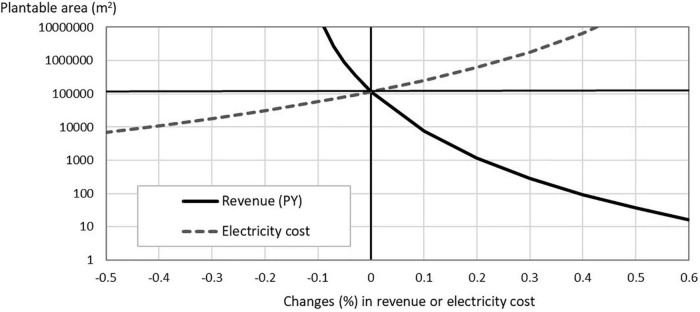
Sensitivity analyses for strawberry production in PFALs: How the minimum plantable area, which satisfies (B/C) = 1 in strawberry production, changes when the revenue (PY) or electricity cost changes (for the above-average PFALs). The starting point before the change is *the minimum plantable area = 115,697 m^2^, Y = 20 kg/m^2^/year, P = US$ 50/kg, and electricity cost = US$ 199/m^2^/year*.

The results are shown in Figure 2 for lettuce and in Figure 3 for strawberries. It is apparent for both crops that changes in revenue give larger impacts on the minimum scale than changes in a cost item. This is because changes in revenue gives larger impacts on “surplus” than changes in one of cost items (see Eq. 5). For lettuce, if the output price declines by 20% (from US $ 11/kg to US $ 8.8/kg), the breakeven minimum scale increases sharply from 38 to 1,700 m^2^. Corresponding to this decline in the lettuce price, the B/C ratio of the investment to build a PFAL of 38 m^2^ declines from 1.0 to 0.8. With this lower lettuce price, the PFAL operator of this small scale PFAL faces to a negative rate of return (-20%/year). Should the lettuce price decline by 35% to US $ 7.2/kg, the breakeven minimum scale would increase to more than 100 ha (Figure 2).

In the case of strawberries, a 20% increase in the unit yield from 20 kg/m^2^/year to 24 kg/m^2^/year by technological advances, or the same rate of increase in the output price because of higher quality of the output, brings about a drastic decline in the breakeven minimum scale from 115,697 to 1,200 m^2^ (Figure 3), which would convert strawberries to economically viable crop to be grown in PFALs.

Changes in production costs, such as labor cost and electricity cost affect the profitability of crops in PFALs and therefore the breakeven minimum scale. Compared to the degree of impact that changes in the revenue give to the profitability, however, the degree of the impact becomes lower as the cost share of the cost item in the total current production cost becomes lower. For example, a 30 % increase in the wage rate increases the breakeven minimum scale from 38 to 110 m^2^ for lettuce (Figure 2). Similarly, a 40% decrease in the electricity cost reduces the minimum scale for strawberries, but only down to 10,000 m^2^ (Figure 3).

## Discussions

### Economies of scale in PFAL construction

The relatively better-quality subset of the main dataset gives the degree of economies of scale (the scale elasticity) in PFAL construction of -0.17 (Figure 1B), with its 95% confidence interval of “-0.12 – -0.22.” Another simplest way to estimate the scale elasticity would be to check the slope of the straight line that connects the PFAL of the smallest size (PFAL #26) and that of the largest size (PFAL #1) in Figure 1B, which is -0.14. All other sample PFALs are located in between the two PFALs, in the neighborhood around the straight line. It would be highly probable that the true value of the scale elasticity is within this 95% confidence interval.

How economies of scale of PFAL construction are compared with those of other industries? The distribution of the scale elasticity (“b” in Eqs 2 and 3) of industrial plant investment estimated by Haldi and Whitcomb (1967) has the mode for the elasticity class of [-0.2 > b > -0.3], with the mean of -0.27. In the case of infrastructure project such as irrigation scheme construction, this elasticity is as large as -0.7 (Inocencio et al., 2007). Comparing to these industries, the scale elasticity of -0.17 is not large. One reason for this observation may be that the PFAL construction cost in this paper includes not only the costs of constructing the factory building but also the costs of installing various facilities and equipment in the factory, such as hydroponic cultivation systems, LED lighting systems, planting racks, etc. These facilities and equipment, though fixed production factors, may be more “divisible” than the factory building, which reduces the degree of scale economies for the total investment.

Though not so large compared to other industries, the mean estimate of the scale elasticity of -0.17 still means that, when the scale of PFALs increases 10, 100, and 1,000 times, say from 100 to 1,000 m^2^, to 10,000, and to 100,000 m^2^, the unit cost of PFAL construction decreases by 33, 55, and 80%, respectively.

### Economically viable crops in PFAL production

A basic implication derived from the results of our exercise is that the list of crops that can be grown commercially in PFALs is very short at present.

It must be clear that grain crops, such as wheat, are out of question. Asseng et al. (2020) concluded in their highly intensive study that wheat production in PFAL of 10 ha scale, or even of 100 ha scale, cannot be economically viable under the present price conditions for wheat and production inputs. It should be noted that their conclusion does not depend on the oft-mentioned barrier to the commercial crop production in PFALs, i.e., “high initial investment cost.” In their case, wheat is not an economically viable crop simply because the current production of wheat in PFALs cannot generate any positive profit, or “surplus” in our Eq. 5: The cost of constructing the PFAL and its scale are nothing to do with their conclusion. In this respect, their wheat case is different from our strawberry case. In the latter case, the current production of strawberries in PFALs can generate “surplus,” but it is not enough to justify the investment to construct a PFAL: The case the “initial investment cost” matters.

Lettuce is an economically viable crop in PFAL production. It should be pointed out again that such a status of lettuce has been established by the yield revolution that occurred in recent years because of improvements in LED technology, new lettuce varieties, and efforts made by PFAL operators to advance lettuce growing technology in PFALs. This point cannot be overemphasized. Until 2017, the unit yield of lettuce in PFAL production in Japan had been less than 60 kg/m^2^/year (see sub-section 2.1 in Supplementary Material). Table 3 in the text, after simple calculations, tells that the unit yield of lettuce less than 67 kg/m^2^/year fails to give any positive surplus. Under such a condition, lettuce is no difference with the wheat case above as far as commercial production in PFALs is concerned: They are equally economically not viable, regardless of the cost of PFAL construction.

The results of our exercise suggest that strawberry production in PFALs under present conditions is not economically viable yet but about to be added to the list of PFAL crops. The sensitivity test shows that a 20% increase in the unit yield transforms it to a crop that is profitably grown. A breakthrough in cultivation technology to increase its yield or to reduce its production costs is expected to come soon, as in lettuce production.

### Vulnerability in crop production in PFALs

The sensitivity tests show that the PFAL’s breakeven minimum scale for lettuce production quickly approaches infinity as the decline in the revenue progresses beyond the decline rate of 30% (Figure 2). This is because the “surplus” approaches zero as the revenue continues to decline. In the case of crop production in PFALs, yield may increase, but not likely to decrease significantly. In contrast, the risk that the output price declines significantly could be high for PFAL operators. Their market outlets are generally not ordinary wholesale markets (see Supplementary Table S7) but direct off-market shipments to supermarkets, ready-to-eat food manufacturers, delicatessens, and restaurants under contracts. To the extent less dependent on the ordinary markets, they face to less risk in seasonal price fluctuations in the markets. Instead, they would have to face to the risk of losing some contracts or requests from contractors for downward price revisions. Such risks could be high because they have many contacts with business partners. In the case of PFAL operators in Japan, on average, each PFAL operator has as many as 30 contracts with buyers, and 30% of them have more than 50 contracts (see Supplementary Table S7). Demand for vegetables of the off-market buyers such as supermarkets, delicatessens, and restaurants may not be subject to seasonal fluctuations, but could be affected by rapidly changing end-consumers’ teste and preference, and these shops and restaurants themselves are operating in tightly competitive markets where the rise and fall of stores and shops is fierce.

The high sensitivities of the rate of return to the investment in PFAL construction to changes in basic parameters related to the crop performance are reported by some past studies. The internal rate of return to the investment on PFAL construction for growing basil ranges wide from 0.04 to 98% among nine scenarios tested (Avgoustaki and Xydis, 2020). Similarly, another case of PFAL construction for growing lettuce, the rate of return to the investment varies among nine scenarios examined from -0.5 to 38% (de Souza et al., 2022). A recent paper by Baumont de Oliveira et al. (2022), assessing financial risk for PFALs, reports that a PFAL actually constructed in the United Kingdom goes bankrupt with *p* = 100% (the case of a negative surplus in the current lettuce production account), if without any intervention, and that the other designed PFAL in Japan (the same PFAL assessed by de Souza et al., 2022 = our PFAL #11) would face, in its 15-year life span, a negative financial balance at about *p* = 30% and below-threshold rates of returns to the investment at about *p* = 50%.

While vegetable production by PFALs is highly touted as “promising,” it is still said to be “hype” (Terazono, 2020; Gordon-Smith, 2021). A reason for this could be the fact that many commercial PFALs have been built so far, many of which have ended up in bankruptcy. As mentioned earlier in this paper, the number of PFALs in Japan has been stagnant since 2017. This does not mean that the PFAL vegetable production industry is in a stationary state. On the contrary, there has been substantial number of new entries in this industry but there has been nearly the same number of exits. Available data suggest that, within a period of 10 years since 2012, nearly 80% of PFALs in Japan have disappeared, being replaced with nearly the same number of new ones (see section 4.2 of Supplementary Material).

One of the most likely causes of this bankruptcy is the high risk associated with the instability in price and contract, which gives serious adverse impacts on profitability. The results of the sensitivity test suggest that lettuce production in PFALs is economically viable, but it is subject to high vulnerability. When planning to establish a PFAL for commercial engagement, it is necessary to have an ample leeway or measures that can sufficiently withstand the risk of instability in output and input prices and in sales and cultivation contracts.

### PFALs’ optimum scale?

The construction of PFALs is subject to economies of scale: The larger the scale, the lower the unit investment cost. As everyone points out, the initial investment cost is the most serious entry barrier. Then, does the scale of PFALs continue to increase to enjoy ever lower initial unit investment costs? In some industries characterized by huge facilities and structures of public nature, such as the power generation and gas supply industries, the scale of plants continues to increase to the extent that the industry becomes a regional monopoly consisting of only one huge company. In many industries, however, this does not occur: The ever-increasing scale is checked at somewhere before this stage. The checking factor is generally called “transaction costs.”

Transaction costs are non-monetary costs that are associated with economic transactions (Williamson, 1979). For example, a PFAL operator has to search if there are someone who buy crops produced by the PFAL, find them out, negotiate with them about the prices and quantities, make contracts, enforce the contracts, and take appropriate actions in case of breach in the contracts: All these actions (transactions) are not costless, or rather often very costly to the PFAL operator. As the number of contractors increases, the transaction costs could increase, even disproportionally. The PFAL operator has to hire laborers and you may have to monitor to have them work carefully with plants. This cost for monitoring and enforcing labor contracts is another example of transaction costs, which would become more costly as the scale of your PFAL becomes larger. Fund markets are prone to market failures, and you may face difficulties in raising funds when planning to construct a PFAL of large scale, which is also a transaction cost.

In Asia, the proliferation of delicatessens, ready-to-eat food manufactures, and restaurants have made themselves good customers of vegetable-producing PFALs. Small- to medium-scale PFALs sell their vegetables, which do not need to be washed before cooking, to many of these customers in small individual packages so that the customers can use them immediately without the hassle of arranging them into small units. As a result, they enjoy a higher unit price for their vegetables than they sell to the ordinary wholesale market. Large-scale PFALs, on the other hand, tend to sell their mass-produced vegetables in bulk to buyers, such as regional centers of large supermarket chains, at low prices. Such a difference should result from high transaction costs associated with dealing with a large number of customers by delivering vegetables in small packaging (often tailor-made for individual customers). The price difference between individual packaging delivery and bulk sales often exceeds 30% (for more details, see sub-section 2.1 of Supplementary Material).

These transaction costs would work to counterbalance the merit of economies of scale. It is an important research agenda to study how these transaction costs and economies of scale in PFAL construction interact, or counteracts, in determining the scale of PFALs and how the optimal scale differs among the type of crops grown and among the various modes of PFAL management.

## Conclusion

In this paper, for the first time in this research field, we have explored whether economies of scale exist in the construction of PFALs and how it affects to the economic viability of vegetable production in PFALs.

We find that economies of scale exist in the costs to construct and set up PFALs. The relatively better-quality subset of our datasets gives the 95% confidence interval of the scale elasticity as “-0.12 to -0.22” with the mean estimate of -0.17. Not a large elasticity compared with other industries, but with this mean elasticity the unit construction cost declines by as much as 55%, to a level less than half, when the scale of PFALs increases 100 times.

The minimum scale, which ensures the breakeven in the crop production in PFALs, is estimated to be less than 40 m^2^ for lettuce and more than 100,000 m^2^ for strawberries, suggesting that the former is an established PFAL crop, but the latter is not yet. The recent revolutionary increase in the yield due to improvements in LED technology, new lettuce varieties, and PFAL operators’ efforts to finetune these new “inputs” toward higher yields has helped lettuce to establish this status. It would be almost certain that such technological advances do occur, sooner or later, for strawberries and other candidate crops for PFAL production.

The breakeven minimum scale is extremely sensitive to changes in the variables to determine the profitability of crop production in PFALs. The impacts of changes in the unit yield of the crop and its price, which together determine the revenue, are particularly large. A 30% decline in the lettuce price puts most lettuce producing PFALs on the brink of bankruptcy, while a 20% increase in the unit yield or the price of strawberries transforms strawberries into an economically viable PFAL crop. This overly sensitive nature of the commercial crop production in PFALs is behind the current state of the crop production in PFALs where many PFALs go bankrupt while being replaced by many newcomers every year. Both pros and cons of PFALs have the grounds for their claims. If you make a claim by looking at only one side of the fact, not knowing or ignoring the other side, however, it could be called “hype.”

The existence of economies of scale may transform the PFAL crop production industry into an industry where large PFALs are more popular than today. However, the emergence of excessively large-scale PFALs would be restricted by transaction costs that are inherent in various aspects of the management of PFAL crop production. Transaction costs typical of the plant factory business would arise from the needs to develop and maintain stable and good-price-offering buyers and to manage the workforce for high-quality plant production. How these transaction costs and economies of scale in PFAL construction interact in determining the optimum scale of PFALs is an important research agenda to be addressed in the future.

## Data availability statement

The sources of all the data used in this article are presented either in the main text or in the Supplementary material. Further inquiries are entertained upon request to the corresponding author.

## Author contributions

MK, YZ, NL, and MT: conceptualization. YZ, MK, SS, and AM: methodology, data preparation, and investigation. MK: writing—rough draft. YZ, MK, NL, AM, and MT: writing—final draft, review, and editing. MK, MT, and NL: supervision. All authors contributed to the article and approved the submitted version.
