# Supplementary material for: Economies of scale in constructing plant factories with artificial lighting and the economic viability of crop production
Source: Front Plant Sci. 2022 Sep 8;13:992194. doi: 10.3389/fpls.2022.992194 (PMC9493372; doi:10.3389/fpls.2022.992194)
Supplement: Supplementary file 1 [file Data_Sheet_1.docx]

Supplementary Material

**Table of contents**

1. Data on PFAL construction cost

1-1 Definitions

1-2 Data collection

1-3 Main dataset

1-4 Auxiliary dataset

1-5 Scale economies with respect to PFALs’ building floor area

2. Lettuce and strawberry production in PFALs

2-1 Production parameters

2-2 Revenue-cost structure

3. Depreciation, maintenance, and interest rate

3-1 Capital depreciation

3-2 Facilities maintenance

3-3 Interest rate as opportunity cost

4. Recent trends and features of commercially operating PFALs in Japan

4-1 Lettuce yield

4-2 Number of PFALs since 2011

4-3 Other features

**1.** **Data on PFAL construction cost**

**1-1 Definitions**

We are interested in PFALs’ minimum scale that ensures the breakeven in the vegetable production in PFALs. We define the breakeven point as the scale at which the benefit-cost (B/C) ratio of the investment for constructing a PFAL becomes unity. For comparing PFALs of different scales, therefore, it is highly convenient and desirable to define the benefits and costs per plantable area of PFALs. First, let us define the PFAL construction cost per plantable area and related variables, as follows:

[a] Factory size = PFAL building floor size ­= the total (ground) floor area of a PFAL,

[b] Cultivation-zone floor area = the floor area taken by planting (cultivation) racks (shelves),

[c] Number of tiers (layers) per planting rack,

[d] Total area for planting = the total plantable area ＝the scale of PFALs = cultivation-zone floor area × the number of tiers per planting rack = [b] × [c],

[e] PFAL building construction cost = the total cost to construct a PFAL building, not including land acquisition cost,

[f] PFAL non-building construction cost = the total cost of acquiring systems for hydroponic plant production, lighting, environment control, planting racks, and all other necessary durable facilities,

[g] PFAL construction cost = PFAL building construction cost + PFAL non-building construction cost = [e] + [f], and

[h] Unit construction cost of PFAL = PFAL construction cost per plantable area = [g]/[d].

Note that we define the scale of a PFAL by its total plantable area [d]. The unit construction cost of the PFAL [h] is obtained by dividing the PFAL construction cost [g] by the total plantable area of the PFAL [d].

This unit construction cost is rarely available as raw data. If some data were available, most popularly, they would be the factory size [a] and the PFAL construction cost [g]. If data on the cultivation-zone floor area [b] and the number of tiers per planting rack [c] are available in addition, we can obtain the unit construction cost of the PFAL in question as [g]/([b]×[c]) = [g]/[d].

Note that we do not include the land acquisition cost in the total construction cost, because this information is the scarcest information among the information related to PFALs’ construction that is very scarce in general. A more basic reason is that the price of land varies greatly among countries, among cities in a country, and among sections in a city. So, the inclusion of the land acquisition cost would disturb the comparison of the PFAL construction cost among countries. If the land acquisition cost were to be accounted for, it would be better to evaluate it in the flow term and include it as a land rent in the current crop production account.

**1-2** **Data collection**

We have searched PFAL construction cost data through the internet. Soon after starting the data collection, we realized that the information on the construction cost of PFALs is awfully scarce, comparing to the information on PFALs in general. In spite of the proliferation of PFALs for commercial crop production, it is rare to see data on detailed specifications of specific PFALs, except for a limited number of monographs and technical books in the field of PFAL research. This scarcity would be due largely to the consideration for protecting technical and other proprietary data, as popularly observed in an emerging industry, and to tax consideration. Even if some information on PFAL construction is available, it is usually very simple one, such as ‘a PFAL of the size of 4000 m^2^ was constructed at the cost of 3 million dollars.’ Many information sources lack some necessary information to obtain the unit construction cost. We have collected data for 40 PFALs from 23 sources. Of these PFALs, only 26 PFALs are provided with necessary information. We use the data on these 26 PFALs obtained from 14 sources, listed in Table 1 in the text, as the main dataset to be analyzed in this paper.

**1-3 Sources of the main dataset**

Supplementary Table S1 shows the sources of 26 PFALs listed in Table 1.

We deigned six PFALs and estimated their construction costs (Source ID #5 in Table 1 and Table S1). These estimated construction costs are presented in Supplementary Table S2.

**1-4 An auxiliary dataset**

One way to check whether the main dataset of 26 observations duly represents the population is to add the dataset some additional observations and see how the results are changed. Since we have data on 14 PFALs for which some data are missing to obtain the scale of PFALs and / or the unit construction cost per plantable area, we try to use these observations while predicting the missing data.

The list of these auxiliary PFALs is shown in Supplementary Table S3. As explained in Foot-note b, the cultivation-zone area [b] is missing for most of auxiliary sample PFALs. The PFAL building construction cost [e] is missing in four data sources. The number of layers per cultivation racks [c] is missing in two data sources.

For sample PFALs for which data on the cultivation-zone area is missing, we try to estimate it by using the relationship between the share of PFALs' cultivation-zone floor area in PFALs' factory floor area and PFALs' factory floor area. Data on this relationship are available for 26 PFALs in the datasets (Fig. S1) and the missing data are estimated by applying the regression equation obtained. Likewise, the construction cost of PFAL building is estimated by applying the relationship between the share of PFALs' building cost in the total construction cost and PFALs' factory floor area (Fig. S2). In case the data on the number of tiers per planting rack is missing, we assume the typical level in the country and the year concerned.

There are a few auxiliary sample PFALs, for which data are given in ranges, such as ‘the construction cost of PFALs of 500 m^2^ to 2000 m^2^ is US$ 1 million to US$ 10 million and that of PFALs ≥ 2000 m2 is ≥ US$ 15 million.’ For such samples, we take the medians for closed ranges and the end point for open ranges.

The results are compared with Fig. 1 [A] of the main text in the following Supplementary Fig. S3. The comparison of the main dataset (N=26) and the (main + auxiliary) datasets (N=40) gives the following observations:

1. The addition of the auxiliary sample PFALs does not alter the significant scale economies that exist in the PFAL construction, although the estimated regression line of the main dataset has a higher intercept and a larger (negative) slope than that of the main + auxiliary dataset (both differences are statistically significant at p < 0.05).
2. The above-average PFALs, which are on and above the regression line, are more in number and smaller in variation, compared to the below-average PFALs, which are less in number and sparsely scattered.
3. For both datasets, the majority of the below-average PFALs form the (bottom frontier) baseline, nearly parallel to but well below the regression line. This baseline may be formed by PFALs that are with relatively lower or simpler technology, and those PFALs on the bottom frontier make the slope and the intercept term lower in the right-hand chart of Fig.S3.

Altogether, the addition of the auxiliary sample PFALs verifies that it would not be inappropriate to assume that the sample PFALs in the main dataset are drawn from the PFAL population. It is also shown that the above-average PFALs in the main dataset would be of good quality PFALs with relatively advanced technology. Although our main dataset is admittingly not of the best quality, we use this dataset for the analyses in this paper, as the second best.

**1-5 Scale economies with respect to PFALs’ building floor area**

In this study, we define the scale of a PFAL as the total plantable area (= cultivation-zone floor area × the number of tiers per planting rack) of the PFAL. We define so, because the total plantable area is the best proxy to represent the scale (= output capacity) of a PFAL. Another possible and easy-to-obtain variable to represent the scale of a PFAL would be PFALs’ building total floor area. It is worth examining how PFALs’ economies of scale would be if we used this variable to measure the scale, instead of the total plantable area.

As shown in Supplementary Fig. S4, PFALs’ total plantable area (A_tp_) is highly correlated with PFALs’ total floor area (A_tf_) in logarithm. It is estimated as follows:

$A_{tp}=c(A_{tf})^{d}= c (A_{tf})^{0.993}$ Eq. [S1]

Note that the slope in the figure is nearly 1.

We define economies of scale for PFAL construction by Eq. [2] in the main text using the PFALs’ total plantable area and estimate this equation as Regression #1 in Table 2 in the main text, as follow:

$$K/{A_{tp}}=a^{'}(A_{tp})^{b}$$

$=a'(A_{tp})^{(-0.201)}$ Eq. [S2]

Insert Eq. [S1] for A_tp_ in the above equation,

$$K/{A_{tp}}=K/[c\left( A_{tf})^{d} \right]=a'[c(A_{tf})^{d}]^{b}=a'c^{b}(A_{tf})^{db}$$

In short,

$K/[c\left( A_{tf})^{d} \right]=a'c^{b}(A_{tf})^{db}$ Eq. [S3]

Multiplying [$c\left( A_{tf})^{d-1} \right]$ both sides of Eq. [S3], we obtain:

$$K/{A_{tf}}=a^{'}c^{b+1}(A_{tf})^{db+d-1}$$

With d = 0.933 and b = – 0.201,

$K/{A_{tf}}=a^{'}c^{b+1}(A_{tf})^{-0.207}$ Eq. [S4]

The scale coefficient for PFALs’ total floor area (A_tf_) is estimated to be – 0.207. The comparison of Eq. [S2] and Eq. [S4] tells that the degree of economies of scale is higher for A_tf_ (PFALs’ total floor area) than for A_tp_ (PFALs’ total plantable area), but the difference is negligible.

Note that such a result is brought about, because the order of differences in area between A_tp_ and A_tf_ is arithmetical, whereas the order of differences in scale is geometrical or logarithmic; as a result, the differences in areas are overwhelmed by the difference in scales.

**2. Lettuce and strawberry production in PFALs**

**2-1 Lettuce**

**Production parameters:** As stated in the main text, lettuce is the most popular crop grown in PFALs. This means that an ample literature is available on the production and cost structure of lettuce. In this Supplementary Materials (SM), we try to identify, based on the past studies and surveys, the level of the unit lettuce yield that PFAL operators with advanced production technology can attain stably.

Supplementary Fig. S5 shows, based on past studies and surveys, what levels of yield, fresh weight, planting density, and planting period are applied and achieved in PFAL lettuce production. In the four histograms in the figure, the mode is found in the ‘30-40 kg /m^2^/year’ class for the yield, in the ‘80-100 g/plant’ class for the fresh weight, in the ‘30-35 plants/m^2^/crop’ class for the planting density, and in the ‘28-30 days’ class for the planting period. Another lower peak is found in the ‘120-140 kg/m^2^/year’ class, in the ‘180-200 g/plant’ class, in the ‘70-100 plants/m^2^/crop’ class, and in the ‘45 days/crop and more’ class, respectively. The modes are formed almost all by experiments conducted by researchers with only one exception, whereas the lower peaks are formed all by PFALs that are commercially operated. It seems that PFAL operators with advanced production technology have significantly outpaced researchers in the productivity performance of lettuce production. Among the data we have collected, the highest yield researchers attain is 69 kg/m^2^/year (Kozai et al., 2018), while a survey conducted by the JGHA (Japan Greenhouse Horticulture Association) reveals that nine PFAL operators in the highest yield class attained, on average, the lettuce yield as high as 169 kg/m^2^/year in 2020. It is worth emphasizing that this improvement in the lettuce production technology of commercially operating PFALs has been a quite recent phenomenon (see Table S5 shown in the Section 4 of this SM).

We understand that two major factors have brought about this revolutionary surge in the yield of lettuce production in PFALs. One is the most recent developments in LEDs, i.e., the wide diffusion of white LEDs, which makes it possible for PFAL operators to increase the amount of light (PPFD = photosynthetic photon flux density) without affecting the temperature, at even cheaper costs than before. The other is the emergence of new lettuce varieties that allow a high planting density in PFAL cultivation. With these technological innovations, PFAL operators can realize higher yields of lettuce through attaining heavier fresh weight per plant and denser planting density, with slightly longer crop durations.

Based on these observations, we assume the following parameters for the lettuce cultivation in PFALs: the fresh weight of 180 g/plant, the planting density of 80 plants/m^2^, the crop duration of 45 days/crop (eight crops per year), which give the unit yield of 115 kg/m^2^/year This is the yield assumed in Table 3 as PFAL operators with advanced production technology can attain stably.

**Revenue-cost-surplus structure:** The price of lettuce and current production costs in Table 3 in the main text, which are assumed based on our experience with the lettuce production in PFALs, are compared with some past studies in Supplementary Table S4. Three Japanese PFAL cases show similar revenue-cost-surplus structures, although de Souza et al. (2022) assumes a lower yield than the level of yield after the ‘yield revolution’ in the late-2010s. Eaves and Eaves (2018) in Canada and Zeidler et al. (2017) in EU assume lower yields and lower prices, yet attaining higher rates of surplus. Although the crop grown in Avgoustaki et al. (2020) is basil, it attains a similar level of surplus rate as our study. For the cost structure, labor takes the largest current production cost item in the 50% of the studies shown, including ours, while electricity is the cost item that takes the highest share in the other 50% of the studies. Particularly, the share of electricity cost is very high in the studies in two EU countries.

**Different market structures among regions:** The higher cost share of electricity in the EU countries may reflect differences in energy prices among countries and regions. It should be remarked that even more important regional differences exist in the markets of PFAL-produced lettuce (Zeidler et al. 2017). In EU and North America, PFAL-produced lettuce is mostly sold in the market where lettuce from greenhouses and open fields is sold together, while in Asia, for example Japan and Singapore, PFAL-produced lettuce can be sold in a separate market where PFAL operators can enjoy higher prices than in the ordinary lettuce market. This separate market consists of buyers, such as delicatessens and other cooked-ready-to-eat-foods makers, who use a large quantity of cut vegetables. Since PFAL-produced lettuce does not need any washing before cooking and if packed into small lots for delivery so that the buyers conveniently use them without any hassle such as sorting, etc., they can offer significantly higher prices than in the ordinary market. Such a difference in the market structure between Asia and EU / North America is reflected in the price differences between these regions observed in Table S4.

This difference in the markets that PFAL operators get access to is applicable even within a country. In Japan, for example, PFAL operators who sell their lettuce to buyers in this separate market tend to be of small- to medium-scale PFALs. For large-scale PFALs, say selling 10,000 plants per day (if the production parameters in Table 3 in the text are applied, the total plantable area of this PFAL is computed as 5,700 m^2^), the PFAL operators incline to sell their produce in bulk to such buyers as supermarkets with large regional franchise. For such buyers, PFAL-produced lettuce may mean nothing more than lettuce grown in greenhouses or open fields, and prices would not make any difference as well. The second PFAL in Table S4 is a large scale one. The lower price received by this PFAL than two other cases in Japan could be due to such a difference.

Note that the share of the packing and distribution cost of our study is disproportionally higher than other cases in Table S4, except for the third case for which this cost share is as high as in our study. This is because the cost includes not only transportation costs for delivering to buyers but also all costs needed for sorting and packing lettuce into some easy-to-handle small sizes. The lettuce price and the packing-distribution costs, therefore, tend to correlate inversely.

**2-2 Strawberry**

Unlike lettuce, strawberry is a crop desired to be cultivated commercially in PFALs but has not been realized yet. This means that there are some experimental studies to cultivate strawberries in PFALs but virtually no study on the revenue-cost structure of its commercial production in PFALs. Even for experimental data by researchers, the available literature is very limited. The data on strawberry production in PFALs presented below are solely for reference purposes and are not claimed to be accurate.

**Production parameters:** Supplementary Fig. S6 shows four histograms based on six studies by researchers. The mode is found of the '5-10 kg/m^2^/year' class for the unit yield, of the '100-250 g/plant' class for the fruit weight, of the '10 plants/m^2^/crop' class for the planting density, and of the ‘120-140 days’ class for the planting period. The highest unit yield reported is 21 kg/m^2^/year, which is brought about by the fruit weight of 814 g/plant, the planting density of 10 plants/m^2^/crop, and the planting period of 140 days/crop (Yoshida et al. 2016). We assume these levels of performance for these production parameters in this study, though it is not known how easy for ordinary PFAL operators to attain these levels of performance.

**Revenue-cost-surplus structure:** Since few data are available on the cost structure of strawberry production in PFALs, we try to estimate the production cost of strawberries by referring to the comparison of the production costs of lettuce and those of tomato, one of fruit vegetables.

First, we assume the labor requirement per crop of strawberry production is twice as much as that of lettuce, referring to Tasgal (2021). Second, the electricity cost for strawberries is assumed to be 20% higher than for lettuce, comparing the experimental data between lettuce and strawberries. Third, for seeds and nutrients (the cost shares of 25:75), we assume the cost is three times more costly for strawberries than for lettuce, per crop for seeds, and per year for nutrients, referring to Tasgal (2021) and da Cunha-Chiamolera et al. (2017). Fourth, we estimate that the cost of packaging and logistics for strawberry is 30% more costly than for lettuce. We also assume there is no difference between the two crops in the water and other costs.

The price of strawberries ranges wide, depending on its quality. In the case of the Japanese market, it ranges from US$ 10/kg to US$ 90/kg. We assume the median price, US $ 50/kg, as the strawberry price.

**Plantable area adjustment:** A serious handicap of strawberries as a crop to be grown in PFALs is that it requires more space for the work passages than lettuce cultivation because of the need to harvest strawberries from both sides of a planting rack. This results in the reduction of the plantable area by, at least, 20%. In table 3 in the main text, which shows the assumed levels of the production performance and the revenue-cost-surplus (profit) structure, the surplus for strawberries is adjusted for this area reduction.

**3.** **Depreciation, maintenance, and interest rate**

To estimate the break-even scale of the PFAL for lettuce and strawberry production using Eq. [8] in the main text, we need to identify the lifespan of PFAL facilities (LS), the maintenance cost rate (α), and the capital interest rate (r).

**Lifespan of the building and facilities of the PFAL (LS):** Asseng et al. (2020) assumes the lifespan of the PFAL building of 20 years. Avgoustaki and Xydis (2020) assumes 20-year lifespan for PFAL facilities, not including the building. Ijichi (2018) assumes 13 years, Tasgal (2019) 15 years, and Zeidler et al. (2017) 25 years, for the PFAL as a whole.

In this paper, we assume the lifespan of 15 years for the PFAL as a whole. This is obtained as the weighted average of the three lifespans: the PFAL building (20 years), the electrical works, air conditioning, hygiene and drainage (15 years), and the cultivation racks and the cultivation environment control system (7 years), using the cost shares of these investment groups (45%, 10%, and 45%, respectively) as weights.

**Maintenance rate (α):** Asseng et al. (2020) assumes the annual maintenance costs explicitly at 0.43% of the total investment. In this study, we assume 1.5% for this rate, which consists of the maintenance of the building (1% of the building construction investments) and of the maintenance of the hydroponic system (1% of the hydroponic system investment), and the replacement of LED lamps (5% of them).

**Capital interest rate (r):** It is difficult to identify an interest rate, because it varies across countries and over time. Asseng et al. (2020) assumes 5%, Avgoustaki and Xydis (2020) 6.25%, Eaves and Eaves (2018) 4.75%, and Zeidler et al. (2017) 3%. In this paper, we assume 5%.

**4. Recent trends and characteristics of commercially operating PFALs in Japan**

The Japan Greenhouse Horticulture Association (JGHA) has been conducting a rare survey of large-scale greenhouses and plant factories, including PFALs, annually since 2010. The reports of this survey, particularly the last five reports, provide us with lots of useful information about PFALs under commercial operation in Japan, not only for setting basic assumptions for the analyses in this paper, as done in the previous section, but also for interpreting and discussing the results of the analyses. Partly because all reports are in Japanese, we present here some data reported in this series of survey reports in the form we tailor-made to fit our purposes to substantiate the results of our study.

**4-1 Lettuce yield**

The unit yield of lettuce grown in PFALs is shown in Table S4. Unfortunately, the data in 2017 might contain some entry errors (see Footnote c to the table). It may be worth reminding that the overall mean yield in 2017 of 28 kg/m^2^/year is close to the mode of lettuce yield in Fig. S4. Taking the data on this overall mean as granted, the unit yield of lettuce in PFALs increases by 2.8 times from 2017 to 2020, or 2.4 times from 2017 to 2021. The mean yield of 169 kg/m^2^/year of PFALs in the highest yield class in 2020 may be subject to some overestimation. There is no doubt, however, that PFALs, whose lettuce unit yield is well over 100 kg/m^2^/year, are not exceptional but layered.

**4-2 Number of PFALs since 2011**

The number of commercially operating PFALs is depicted in Fig. S6 for 2017 – 2022 and the distribution of 46 PFALs operating as of March 2022 by their founding year is summarized in Table S6.

It is apparent that the number of commercially operating PFALs increased rapidly from about 60 in 2011 to about 190 in 2015 and has stagnated since then at about the 190 PFAL-line. This stagnation does not mean a steady state, but, rather, a kind of turbulence is hidden behind it. Table S6 indicates that nearly 80% of the 46 PFALs responding to this question launched their PFAL business in the last 10 years, and on average 3.6 PFALs have kept entering this business every year. If this sample of 46 PFALs represents well the population of about 190 PFALs, 3.6 PFALs means 15 PFALs in the population. Fig. S6 and Table S5 together imply that 15 PFALs entered the industry and another 15 PFALs exited every year since 2015. For 10 years, 150 PFALs. This set of data suggests that within a period of 10 years nearly 80% of PFALs (150/190) disappear and are replaced by new PFALs.

**4-3 Other features**

Using data in the latest report (JGHA 2022), Table S7 summarizes the distribution of PFALs by cultivating crop, shipping destination, the number of destinations, and balance in crop production.

**Cultivating crop** (A): It is confirmed that an overwhelming majority of PFALs cultivate lettuce. A few PFALs cultivate strawberries.

**Shipping destination** (B): The major shipping destination of PFALs is off-ordinary markets.

**Number of destinations** (C): On average, a PFAL operator sell his/her crop product to more than 30 buyers, and 30% of them deal with more than 50 buyers.

**Balance of revenue and cost** (D): Nearly 70% of PFALs are recording deficits in their commercial crop production. If look at PFALs of smaller scale (less than 1000 m^2^ of plantable area), nearly 80% of PFALs are suffering losses. For PFALs of larger scale (1000 m^2^ or larger), the percentage share of deficit PFALs is greatly reduced, but the ratio of surplus PFALs to deficit PFALs is still 50:50.

**References**

Alberta Agriculture and Forestry. 2021. Vertical Farming Case Study. <https://open.alberta.ca/dataset/1c1f48a2-63b9-4dcb-823c-aa49a9a7c810/resource/b0cdfe26-7058-4ee9-a40a-7a3dcff2fe89/download/af-vertical-farming-case-study-2021-04.pdf> (Accessed 19 November 2021)

Alibaba. 2022. Alibaba.com. <https://www.alibaba.com/product-detail/Hydroponic-vertical-plant-factory-40ft-container_1600118796279.html?spm=a2700.7724857.normal_offer.d_image.2e38243avbl9Mj> (Accessed 12 Jan. 2022)

Amidi-Abraham, D. 2021. Understanding capital expenses for vertical farms and greenhouse. AgriTecture. https://www.agritecture.com/blog/2021/1/25/understanding-capital-expenses-for-vertical-farms-and-greenhouses (Accessed 3 Jan. 2022)

A-Plus. 2020. Farm & Factory TAMURA Plant Factory. <https://www.a-plus-tamura.com/farm-factory> (Accessed on 2 Jan. 2022)

Asseng et al. 2020, Wheat yield potential in controlled-environment vertical farms, *PNAS* 117 (32) 19131- 19135, https://www.pnas.org/content/pnas/117/32/19131.full.pdf (Accessed 19 Nov. 2021)

Avgoustaki, D.D., and Xydis, G. 2020. Indoor vertical farming in the urban nexus context: Business growth and resource savings. *Sustainability*, 12, 1965 doi:10.3390/su12051965

Cambridge HOK. 2021. How much does vertical farming cost? <https://cambridgehok.co.uk/news/how-much-does-vertical-farming-cost> (Accessed on 3 Jan. 2022)

Chagvardieff, P., d'Aletto, T., and Andre, M. 1994. Specific effects of irradiance and CO_2_ concentration doublings on productivity and mineral content in lettuce. *Advances in Space Research*, 14(11), 269-275.

Choi, H. G., Moon, B. Y., & Kang, N. J. 2015. Effects of LED light on the production of strawberry during cultivation in a plastic greenhouse and in a growth chamber. *Scientia Horticulturae*, 189, 22-31.

da Cunha-Chiamolera, T.P.L., Urrestarazu, M., Filho,A.B.C., and Morales, I. 2017. Agronomic and economic feasibility of tomato and lettuce intercropping in a soilless system as a function of the electrical conductivity of the nutrient solution. *HortScience* 52(9):1195–1200. doi: 10.21273/HORTSCI12170-17

Eaves, J., and Eaves, S. 2018. Comparing the profitability of a greenhouse to a vertical farm in Quebec. *Canadian Journal of Agricultural Economics* 66: 43-54. DOI: 10.1111/cjag.12161

Hayashi, E. 2020. Selected PFALs in Japan, in Kozai, T., et al. eds., *Plant factory: An indoor vertical farming system for efficient quality food production*, Academic Press, pp.437-454.

Ijichi, H. 2018. Plant factory business: Today and tomorrow of plant factory management. Chapter 3 of Nomura agri-planning & advisory ed., *NARA Report 2018*, Nomura Holdings, pp. 58-80 (in Japanese). <https://www.nomuraholdings.com/jp/company/group/napa/data/20180219_03.pdf> (Accessed 2 Jan. 2020)

Ishinomaki City (Miyagi Prefecture, Japan) 2010. Plant Factory and Costs, Chapter 4 of *Ishinomaki City Agri-Cluster Basic Plan*, pp. 61-72 (in Japanese). https://www.city.ishinomaki.lg.jp/cont/10451000/6544/004.pdf (Accessed 19 Nov. 2021; although this URL is off, we keep its hard copy.)

Japan Greenhouse Horticulture Association (JGHA). 2016, 2017, 2018, 2019, 2020, 2021, 2022. Survey and case report on large-scale greenhouses and plant factories (each-year edition) (in Japanese). <https://jgha.com/dl/> (Accessed 1 March 2022)

Joshi, J., Zhang, G., Shen, S., Supaibulwatana, K., Watanabe, C. K., & Yamori, W. 2017. A combination of downward lighting and supplemental upward lighting improves plant growth in a closed plant factory with artificial lighting. *HortScience,* 52(6), 831-835.02

Kozai, T. 2013. Plant factory in Japan: Current situation and perspective, *Chronica Horticulturae* 53 (2): pp.8-11.

Kozai, T., Hayashi, E., and Amagai, Y. 2018. Plant factories with artificial lighting (PFALs) toward sustainable plant production. In XXX International Horticultural Congress IHC2018: II International Symposium on Soilless Culture and VIII International 1273 (pp. 251-260).

Kozai, T., and Niu, G. 2019. Role of the plant factory with artificial lighting (PFAL) in urban areas, in Kozai, T., et al. eds. *Plant Factory: An Indoor Vertical Farming System for Efficient Quality Food Production*, Academic Press, pp. 7-34.

Li, L., Tong, Y. X., Lu, J. L., Li, Y. M., and Yang, Q. C. 2020. Lettuce growth, nutritional quality, and energy use efficiency as affected by red–blue light combined with different monochromatic wavelengths. *HortScience,* 55(5), 613-620.

Lu, N., Kikuchi, M., Keuter, V., and Takagaki, M. 2022. Business model and cost performance of mini-plant factory in downtown, in Kozai, T., et al. eds. *Plant Factory: Basics, Applications and Advances,* Academic Press, pp. 271-293. <https://doi.org/10.1016/B978-0-323-85152-7.00002-1>

Maeda, K., and Ito, Y. 2020. Effect of different PPFDs and photoperiods on growth and yield of everbearing strawberry ‘Elan’in plant factory with white LED lighting. *Environmental Control in Biology*, 58(4), 99-104.

Markets and Markets. 2021. Market Report: Plant factory market by growing system (soil-based, non-soil-based, and hybrid), facility type (greenhouses, indoor farms, other facility types), light type, crop type (vegetables, fruits, flowers & ornamentals), and region - global forecast to 2026. <https://www.marketsandmarkets.com/Market-Reports/plant-factory-market-199919959.html> (Accessed 3 Jan. 2022)

Nicole, C. C. S., Charalambous, F., Martinakos, S., Van De Voort, S., Li, Z., Verhoog, M., & Krijn, M. 2016 (May). Lettuce growth and quality optimization in a plant factory. In VIII International Symposium on Light in Horticulture 1134 (pp. 231-238).

Ohyama, K. 2015. Actual management situation of large-scale PFAL operation. *Facilities and Horticulture*, No. 168: 30-33 (in Japanese).

Ohyama, K., Yamaguchi, J., & Enjoji, A. 2018. Evaluating labor productivity in a plant production system with sole-source lighting: A case study. *HortTechnology*, 28(2), 121-128.

Ohyama, K., Yamaguchi, J., & Enjoji, A. 2020. Resource utilization efficiencies in a closed system with artificial lighting during continuous lettuce production. *Agronomy*, 10(5), 723.

Pennisi, G., Orsini, F., Blasioli, S., Cellini, A., Crepaldi, A., Braschi, I., ... & Marcelis, L. F. 2019. Resource use efficiency of indoor lettuce (Lactuca sativa L.) cultivation as affected by red: blue ratio provided by LED lighting. *Scientific Reports*, 9(1), 1-11.

Saengtharatip, S., Lu, N., Takagaki, M., & Kikuchi, M. 2018. Productivity and cost performance of lettuce production in plant factory using various light-emitting-diodes of different spectra. *ISSAAS Journal*, 24, 1-9.

Tasgal, P. 2019. The economics of local vertical & greenhouse farming are getting competitive. <https://agfundernews.com/the-economics-of-local-vertical-and-greenhouse-farming-are-getting-competitive> (Accessed 15 Jan. 2022)

Tasgal, P. 2021. Behind the greens: Why greenhouse lettuce is not competitive yet. https://agfundernews.com/behind-the-greens-why-hydroponic-lettuce-is-not-competitive-yet (Accessed March 25, 2022)

Uraisami, K. 2022. How to integrate and to optimize productivity. In: Plant Factory Basics, Applications and Advances. Elsevier, pp 217–249. <https://doi.org/10.1016/B978-0-323-85152-7.00024-0>

Yanan, W. 2018. Plant factories, born from environmental and food safety issues, are advanced stages in the development of protected agriculture, and development trends and driving factors of modern agriculture (in Chinese). https://www.qianzhan.com/analyst/detail/220/181008-6ad6f336.html#comment (Accessed 28 March 2022).

Yoshida, H., Hikosaka, S., Goto, E., Takasuna, H., & Kudou, T. 2012. Effects of light quality and light period on flowering of everbearing strawberry in a closed plant production system. In VII International Symposium on Light in Horticultural Systems 956 (pp. 107-112).

Yoshida H, Mizuta D, Fukuda N, et al. 2016. Effects of varying light quality from single-peak blue and red light-emitting diodes during nursery period on flowering, photosynthesis, growth, and fruit yield of everbearing strawberry[J]. *Plant Biotechnology,* 33(4): 267-276.

Yu, J., Wang, M., Dong, C., Xie, B., Liu, G., Fu, Y., Liu, H. 2015. Analysis and evaluation of strawberry growth, photosynthetic characteristics, biomass yield and quality in an artificial closed ecosystem. *Scientia Horticulturae*, 195, 188-194.

Zeidler C, Schubert D, Vrakking, V. 2017. Vertical farm 2.0: Designing an economically feasible vertical farm-A combined European endeavor for sustainable urban agriculture. https://elib.dlr.de/116034/. Accessed 8 April 2022
